# 3D-guided cadaveric fibula reconstruction for pediatric infratemporal solitary fibrous tumor

**DOI:** 10.1080/23320885.2025.2560394

**Published:** 2025-09-16

**Authors:** Jacob Beiriger, Nilam D. Patel, Hilary C. McCrary, Richard B. Cannon

**Affiliations:** Department of Otolaryngology – Head and Neck Surgery, University of Utah, Salt Lake City, UT, USA

**Keywords:** Pediatric solitary fibrous tumor, 3D surgical planning, zygomatic reconstruction

## Abstract

A fifteen-year-old with infratemporal myxoid solitary fibrous tumor underwent en bloc parotid–infratemporal–extradural skull-base resection. Her zygoma was reconstructed using 3D-planned cadaveric fibula allograft and custom plate. At 6 months she maintained facial symmetry and function. Her case demonstrates digital planning, cadaveric bone, and microsurgical adaptability.

## Introduction

Solitary fibrous tumors (SFTs) are rare mesenchymal neoplasms that can be problematic. The potential for local invasion and overlap with other spindle cell tumors both contribute to diagnostic and therapeutic difficulty [1,2]. Tumors in the infratemporal fossa are often difficult to access due to depth and proximity to neurovasculature [3]. The differential diagnosis for an infratemporal mass includes rhabdomyosarcoma, neuroblastoma, lymphatic malformations, juvenile nasopharyngeal angiofibroma and schwannoma in pediatric patients. Imaging characteristics, location and patient age can narrow differentials, but biopsy is used for definitive diagnosis. We present a pediatric case of an infratemporal SFT managed with en bloc skull base resection, 3D-guided zygomatic reconstruction and microvascular soft tissue coverage.

## Patients/materials and methods

**Figure 1.**
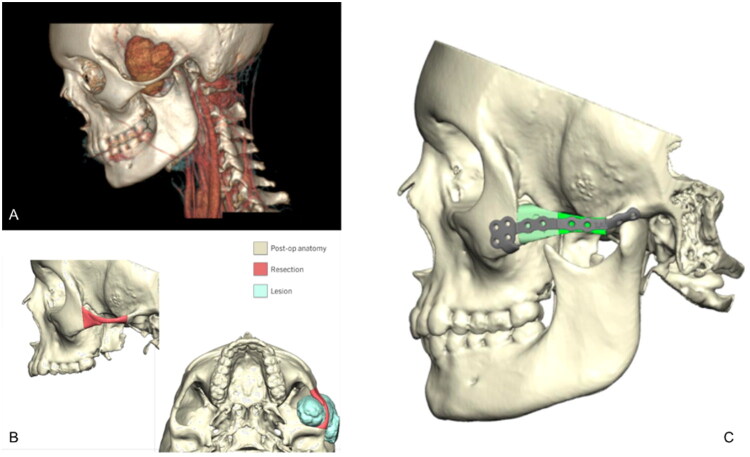
Preoperative imaging and virtual surgical planning. (A) 3D reconstructed imaging demonstrating the left infratemporal fossa with erosion and thinning of the zygoma. (B) Lateral view and inferior view of 3D-rendered skull models demonstrate the extent of the planned maxillary and zygomatic resection (red) and the tumor lesion (blue). Postoperative anatomy is rendered in tan. Virtual planning enabled precise contour matching and resection margin prediction prior to surgery. (C) A digitally rendered lateral skull view demonstrates the placement of a two-segment cadaveric fibula allograft (green) contoured with a 3D-printed cutting guide to restore zygomatic symmetry. The graft is secured with a custom midface titanium plate spanning the reconstructed zygomatic arch.

A 15-year-old female presented with a painless and enlarging left temporal mass. MRI revealed a 5.4 cm lesion in the infratemporal fossa with zygomatic remodeling and skull base contact (Figure 1(A)). Core biopsy confirmed a myxoid variant SFT and tumor board evaluation determined surgical excision would provide the best care. Surgical access was achieved with a preauricular and coronal approach. En bloc resection included a left total parotidectomy, removal of the zygomatic arch, infratemporal and parapharyngeal dissection, and extradural skull base resection.

**Figure 2.**
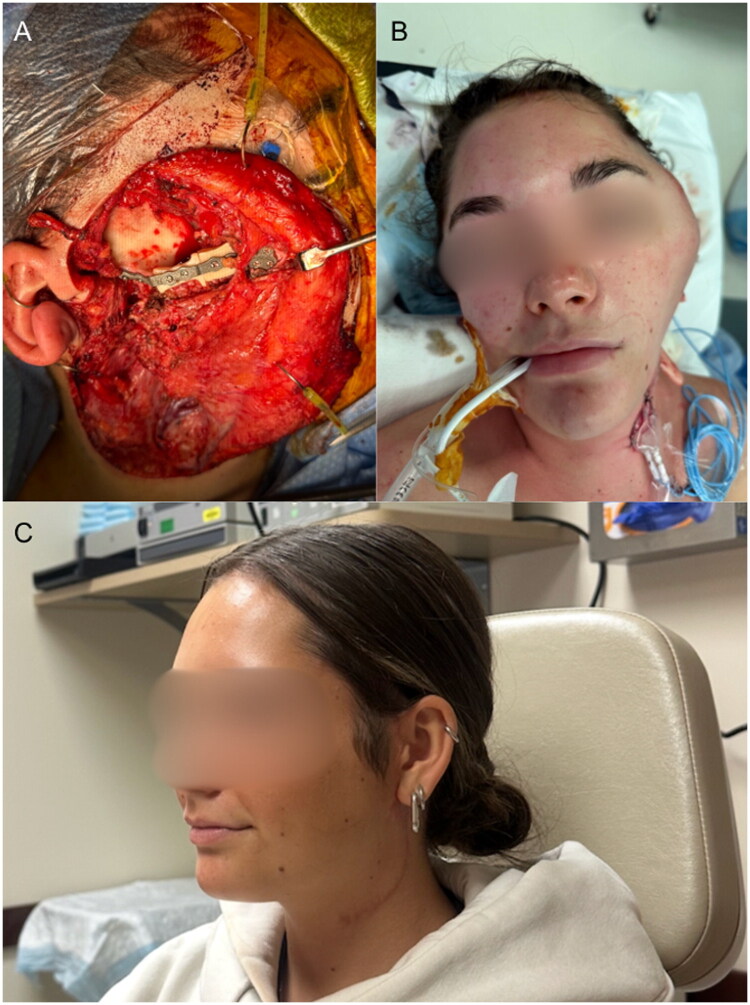
Intraoperative and postoperative imaging. (A) Intraoperative image of the two-segment cadaveric fibula graft with the custom plate, recreating and mirroring the contralateral zygoma to restore midface projection. (B) Immediate postoperative outcome with vascularized anterolateral thigh free flap covering the zygoma reconstruction and primary closure of the skin. (C) Final postoperative outcome three months after surgery.

A cadaveric fibula graft was contoured to reconstruct the midface. A 3D-printed cutting guide was used to segment the fibula into two portions and mirror the zygomatic arch (Figure 1(B,C)). The graft was fixated with a custom titanium plate (Figure 2(A)). A left anterolateral thigh (ALT) flap was used for soft tissue coverage, and the skin paddle was discarded due to bulk. After the operation, she was managed in the ICU with hourly vital signs and hourly flap checks. Early postoperative hemodynamics were stable without vasopressor requirement. On postoperative day 5, there was loss of Doppler signal. This prompted re-exploration, revealing complete flap thrombosis and failure. A new contralateral muscle-only ALT flap was harvested and inset, with new anastomosis to the external carotid artery and a branch of the internal jugular vein. No malocclusion was recorded following the right ALT or at follow-up. Clinical examination and subjective history did not suggest coronoid impingement against the ALT flap, and no occlusal adjustments, elastics, debulking or coronoidectomy were required.

At three-month follow up, she had full facial symmetry and function, normal diet and weight, and reported a mild, but improving, ‘first-bite’ zinging sensation. CT neck with contrast demonstrated no residual or recurrent disease and no reconstruction-related complication. Donor sites were noted to have healed appropriately, strength and ambulation were normal, and she returned to athletic activity.

### Ethical approval

No ethical approval was required per the University of Utah Institutional Review Board (IRB: #00045048).

### Consent for publication

Written informed consent was obtained from both the patient and her legal guardian for publication and is on file with the University of Utah.

### Consent for photographs

Written informed consent was obtained from the participant for publication of identifying details and clinical images. All efforts have been made to ensure patient anonymity.

## Results

Past medical history was otherwise unremarkable; she denied tobacco, alcohol and recreational drug use, and she had no known allergies. No chronic home medications were recorded prior to surgery. At discharge and outpatient follow-up, the patient used acetaminophen as needed, aspirin for 24 hours, ocular lubrication to the left eye, celecoxib as needed, and multivitamins; none were expected to interfere with wound healing or flap perfusion.

The patient experienced flap thrombosis on postoperative day 5, requiring emergent revision with a new contralateral muscle-only ALT flap. She remained hospitalized for 11 days. During hospitalization she had a staged recovery involving the ICU, return to the OR, and gradual return to normal activity. She recovered without further incident and was discharged in stable condition. Final pathology confirmed a 5.4 × 4.0 × 1.9 cm myxoid SFT with negative margins, 4 mitoses per 10 HPF, and no necrosis or lymphovascular invasion. All margins were negative for tumor. She was discharged without complications and preserved facial nerve function. At three-month follow-up, she resumed full activity with full facial symmetry and no signs of recurrence (Figure 2(C)). At six months, she remained disease-free with stable imaging, excellent functional recovery and durable graft viability.

## Discussion

Solitary fibrous tumors of the head and neck account for less than 2% of all soft tissue tumors in the head and neck [4]. SFTs, often indolent, can display aggressiveness based on histologic subtype, location and margins of resection [1]. Myxoid variants of SFTs can cause local mass effects in deep anatomic compartments like the infratemporal fossa [2]. This poses diagnostic and therapeutic challenge for the surgeon due to variable immunohistochemical profiles and proximity to vital neurovascular structures [2, 3].

Surgical excision with negative margins remains the mainstay of treatment. It is important to assess difficulty of each case as SFTs can extend to the skull base, orbit or deep facial skeleton and access may be formidable. Recurrence can also be an issue. Marti-Flich et al. found that positive margins were the strongest predictor of recurrence regardless of histologic grade [5]. A national database analysis by Abiri et al. further found margin status to be independently correlated with survival [4].

The SFT in this case abutted the skull base which required the surgical team to coordinate excision and subsequent reconstruction. Using a cadaveric fibula allograft with a 3D-printed cutting guide allowed us to reconstruct the zygoma with excellent aesthetic outcomes. We mention that, while not the standard, cadaveric bone may be appropriate in skeletally immature patients not expected to undergo postoperative radiation. Vascularized flaps offer durability, however, one in five pediatric patients will require revision surgery [6]. Vascularized flaps may pose a surgical burden in younger patients. We also want to mention the use of alloplastic materials like titanium or porous polyethylene. For now, their role remains limited in pediatric patients because of concerns for growth restriction, extrusion risk and extended integration [7].

Our group wants to emphasize the importance of balancing long-term growth potential, aesthetic symmetry and surgical burden when choosing flap type. Fresh bone grafts have lower infection and failure rates than synthetic implants and banked grafts are associated with increased resorption and complication rates [8]. The osteocutaneous fibula remains the standard for many cases requiring bony and soft tissue restoration and is often found in oncologic cases where defects involve both loss of structure and volume. Fibula flaps offer single-stage reconstruction and also immediate dental restoration. A recent meta-analysis involving pediatric microvascular fibula flaps demonstrated a 96% survival rate and a 9% infection rate [9]. Our case had a rare scenario of early ALT failure in a non-irradiated pediatric patient. Arterial thrombosis occurred on day 5 but revision with a contralateral ALT flap was able to preserve the allograft and facial aesthetic. While flap compromise in ALT reconstructions is uncommon, rates of 3–13% have been reported in pediatric literature [10].

Age and skeletal maturity act as determinants in graft selection for pediatric bone reconstruction. When skeletally immature, vascularized autologous fibula grafts are preferred for long bone segments. This is even more so the case when living bone is required for growth, dental rehabilitation, or when adjuvant radiation is expected. This technique supports long-term function, allows for hypertrophy, and adapts to craniofacial growth [11–15]. Further, the fibula graft can integrate with the growing skeleton, and the proximal epiphysis may actually preserve growth potential in select cases [16–18]. Donor-site morbidity should also be considered in younger children: ankle valgus and instability are more frequent in patients under mid-adolescence. Preservation of the distal fibular segment and distal tibiofibular synostosis can reduce risks, and we see generally favorable functional outcomes [11, 15, 18]. Skeletal maturity should be assessed using left hand and wrist radiographs with Greulich–Pyle standards and standing leg, while ankle radiographs are used to document open growth plates and guide osteotomy planning to preserve the distal fibula [11, 18]. Cadaveric or allograft bone helps to avoid donor-site morbidity but lacks growth potential; it is also associated with higher rates of resorption and failure in children. We find it is most suitable for small, non-load-bearing defects where future remodeling or dental implantation is not required [14].

Our patient’s tumor demonstrated low mitotic activity and negative margins which placed her in a low-risk category [1]. The deep tumor location and complexity of resection are associated with increased recurrence even in histologically indolent lesions [5]. Surveillance was therefore warranted, especially when considering reports of recurrence occurring more than 36 months after resection [1]. Mean time to recurrence was 67 months in a review of all head and neck SFTs reinforcing extended surveillance beyond three to five years in cases with deep location or close margins [5]. Imaging recommendations favor MRI due to soft tissue contrast. Serial imaging should be repeated every 6–12 months for 3 years and followed with annual monitoring through years 3–7. In pediatric patients we recommend to consider longitudinal factors like bone remodeling, psychosocial development and functional restoration. These are areas that are not well explored in the literature. Our study is limited by short duration of follow-up (6 months) which restricts evaluation of long-term graft viability and general outcomes.

## Conclusions

This case touches on digital planning in pediatric facial reconstruction, cadaveric bone in midface repair, and intraoperative adaptability. It also emphasizes the value of longitudinal, multidisciplinary care in young patients with rare skull base tumors.

## Data Availability

This case report does not involve a dataset generated or analyzed beyond the patient’s medical record. Due to patient privacy and institutional policies, the data supporting the findings of this study are not publicly available. De-identified data may be made available from the corresponding author upon reasonable request and with appropriate institutional approvals.
